# 1-Bromo-3,5-diphenyl­benzene

**DOI:** 10.1107/S1600536809045061

**Published:** 2009-11-14

**Authors:** Zhi-Qiang Wang, Hong-Mei Li, Xiao-Juan Sun, Fei-Fei Cen, Bao-Ming Ji

**Affiliations:** a College of Chemistry and Chemical Engineering, Luoyang Normal University, Luoyang 471022, People’s Republic of China; b Library of Luoyang Normal University, Luoyang 471022, People’s Republic of China; c Liming Research Institute of Chemical Industry, Luoyang 471001, People’s Republic of China

## Abstract

The title compound, C_18_H_13_Br, crystallizes with two crystallographically independent mol­ecules in the asymmetric unit. The C—Br bond lengths and the C—C bond lengths between the benzene rings are slightly different in the two mol­ecules. The dihedral angles between adjacent benzene rings are 26.85 (2) and 39.99 (2)° in one mol­ecule, and 29.90 (2) and 38.01 (2)° in the other. There are three types of inter­molecular C—H⋯π inter­actions in the crystal structure.

## Related literature

For blue light-emitting diodes based on 3,5-diaryl-phenyl derivatives, see: Niu *et al.* (2004 ▶). For the synthesis of the title compound, see: Kim *et al.* (2001 ▶). For the importance of C—H⋯π contacts and their geometries, see, for example: Suezawa *et al.* (2004 ▶).
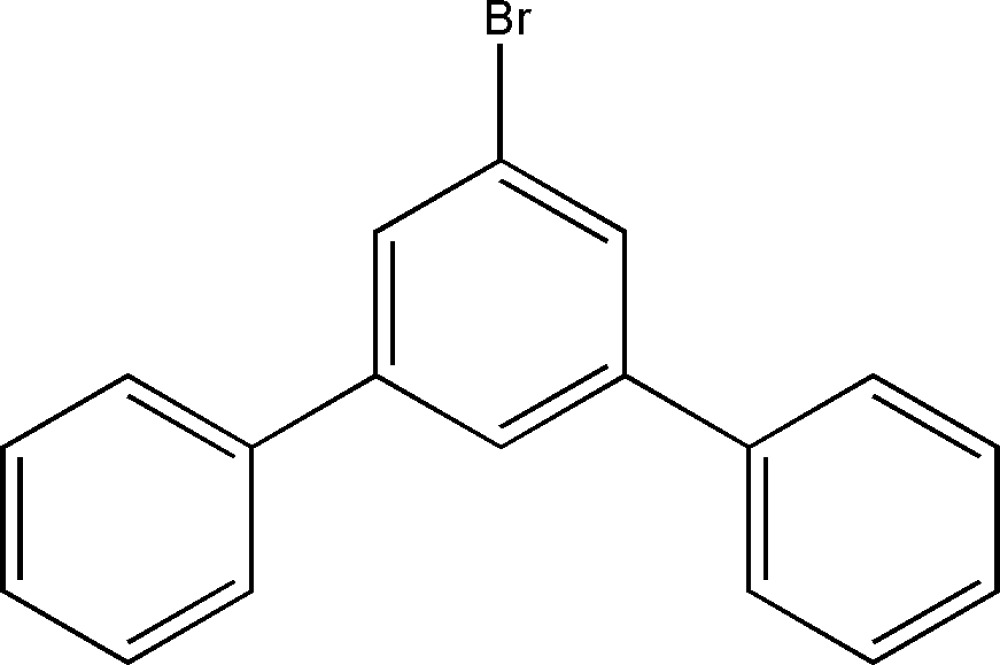


## Experimental

### 

#### Crystal data

C_18_H_13_Br
*M* *_r_* = 309.19
Monoclinic, 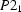

*a* = 11.0782 (12) Å
*b* = 7.7495 (8) Å
*c* = 16.7782 (17) Å
β = 107.441 (1)°
*V* = 1374.2 (2) Å^3^
*Z* = 4
Mo *K*α radiation
μ = 2.97 mm^−1^
*T* = 294 K
0.41 × 0.13 × 0.09 mm

#### Data collection

Bruker SMART APEX CCD area-detector diffractometer
Absorption correction: multi-scan (*SADABS*; Sheldrick, 1996 ▶) *T*_min_ = 0.375, *T*_max_ = 0.776
7808 measured reflections
4775 independent reflections
4138 reflections with *I* > 2σ(*I*)
*R*_int_ = 0.014

#### Refinement

*R*[*F* ^2^ > 2σ(*F* ^2^)] = 0.030
*wR*(*F* ^2^) = 0.076
*S* = 1.00
4775 reflections
343 parameters
1 restraint
H-atom parameters constrained
Δρ_max_ = 0.23 e Å^−3^
Δρ_min_ = −0.31 e Å^−3^
Absolute structure: Flack (1983 ▶), 2006 Friedel pairs
Flack parameter: 0.007 (7)

### 

Data collection: *SMART* (Bruker, 2004 ▶); cell refinement: *SAINT* (Bruker, 2004 ▶); data reduction: *SAINT*; program(s) used to solve structure: *SHELXS97* (Sheldrick, 2008 ▶); program(s) used to refine structure: *SHELXL97* (Sheldrick, 2008 ▶); molecular graphics: *SHELXTL* (Sheldrick, 2008 ▶); software used to prepare material for publication: *SHELXL97* and *PLATON* (Spek, 2009 ▶).

## Supplementary Material

Crystal structure: contains datablocks global, I. DOI: 10.1107/S1600536809045061/si2218sup1.cif

Structure factors: contains datablocks I. DOI: 10.1107/S1600536809045061/si2218Isup2.hkl

Additional supplementary materials:  crystallographic information; 3D view; checkCIF report

## Figures and Tables

**Table 1:** Hydrogen-bond geometry (Å, °)

*D*—H⋯*A*	*D*—H	H⋯*A*	*D*⋯*A*	*D*—H⋯*A*
C15—H15⋯*Cg*1^i^	0.93	2.82	3.601 (4)	142
C18—H18⋯*Cg*6^ii^	0.93	2.84	3.682 (4)	152
C20—H20⋯*Cg*1^iii^	0.93	2.92	3.603 (4)	132

Symmetry codes: (i) 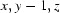; (ii) 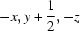; (iii) 
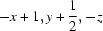
. *Cg*1 and *Cg*6 are the centroids of the C19–C24 and C13–C18 benzene rings, respectively.

**Fig. 1.**
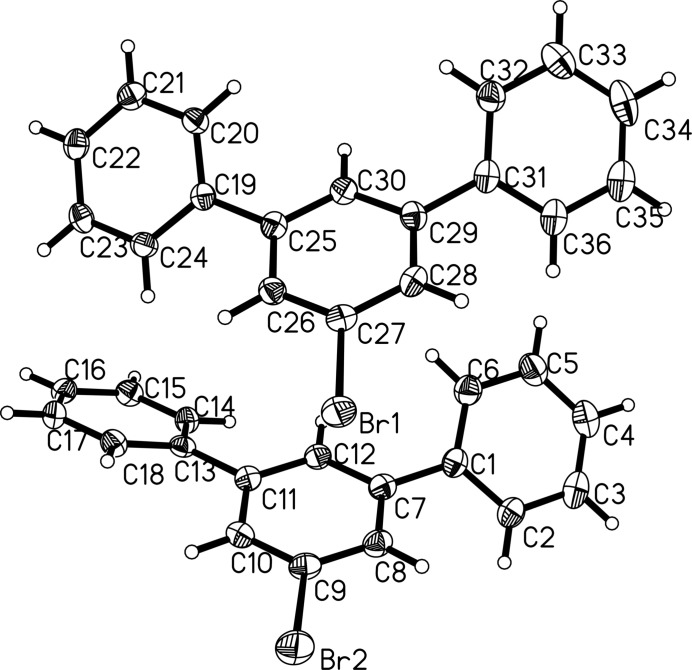
The two independent molecules of the title compound with labelled non-hydrogen atoms using 30% probability ellipsoids.

## supplementary crystallographic information

### Comment

3,5-Diphenylbenzene is a good substituent for organic light-emitting materials. The thermal properties, photophysical properties and film formation properties of organic light-emitting materials can be improved efficiently by introducing 3,5-diphenylbenzene group (Niu, *et al.*, 2004). The title compound, 3, 5-Diphenyl-1-bromobenzene, is generally used as the precursor to produce the 3, 5-diphenylbenzene group by Suzuki coupling reaction. The crystal structure of the title compound has not been reported to the best of our knowledge.

A view of the two independent molecules of the title compound shows distinct rotations of phenyl rings (Fig.1). The dihedral angles between adjacent benzene rings are 26.85 (2)° and 39.99 (2)° in one molecule, 29.90 (2)° and 38.01 (2)° in the other molecule. The C—Br bond lengths in the two crystallographically independent molecules are 1.903 (3) Å and 1.911 (3) Å, and the C—C bond lengths between benzene rings are 1.480 (5) Å (C1—C7), 1.486 (4) Å (C11—C13), 1.491 (4) Å (C19—C25), and 1.484 (4) Å (C29—C31), respectively. Three types of weak intermolecular C—H···π interactions exist in the crystal structure (Table 1, *Cg*1 and *Cg*6 are the centroids of the benzene rings C19 - C24 and C13 - C18). A detailed discussion for C—H···π interactions and geometries was presented by Suezawa *et al.* (2004).

### Experimental

The title compound was synthesized according to the reported procedure (Kim, *et al.*, 2001). A mixture of phenylboronic acid (0.49 g, 4 mmol), 1,3, 5-tribromobenzene (0.63 g, 2 mmol) and Pd(PPh_3_)_4_ (20 mg) was added 20 ml tetrahydrofuran and 8 ml aqueous potassium carbonate (2 *M*). The mixture was vigorously refluxed under a nitrogen atmosphere. The reaction mixture was allowed to cool to room temperature, and then extracted by ethyl acetate. The organic layer was dried over anhydrous magnesium sulfate. After removing the solvent in vacuum, the crude product was purified by column chromatography using petroleum ether as eluent. The title compound was obtained in 54% yield. The single crystals suitable for the X-ray crystallographic analysis were obtained by slow evaporation of a dichloromethane solution as colorless blocks.

### Refinement

All of the H atoms were positioned geometrically with C—H of 0.93 Å and were constrained in a riding motion on their parent carbon atoms with *U*_iso_(H) = 1.2*U*_eq_(C).

### Crystal data

**Table:**

C_18_H_13_Br	*F*(000) = 624
*M**_r_* = 309.19	*D*_x_ = 1.494 Mg m^−^^3^
Monoclinic, *P*2_1_	Mo *K*α radiation, λ = 0.71073 Å
*a* = 11.0782 (12) Å	Cell parameters from 3281 reflections
*b* = 7.7495 (8) Å	θ = 2.7–24.8°
*c* = 16.7782 (17) Å	µ = 2.97 mm^−^^1^
β = 107.441 (1)°	*T* = 294 K
*V* = 1374.2 (2) Å^3^	Block, colourless
*Z* = 4	0.41 × 0.13 × 0.09 mm

### Data collection

**Table:**

Bruker SMART APEX CCD area-detector diffractometer	4775 independent reflections
Radiation source: fine-focus sealed tube	4138 reflections with *I* > 2σ(*I*)
graphite	*R*_int_ = 0.014
φ and ω scans	θ_max_ = 25.5°, θ_min_ = 2.7°
Absorption correction: multi-scan (*SADABS*; Sheldrick, 1996)	*h* = −13→13
*T*_min_ = 0.375, *T*_max_ = 0.776	*k* = −9→8
7808 measured reflections	*l* = −20→19

### Refinement

**Table:**

Refinement on *F*^2^	Secondary atom site location: difference Fourier map
Least-squares matrix: full	Hydrogen site location: inferred from neighbouring sites
*R*[*F*^2^ > 2σ(*F*^2^)] = 0.030	H-atom parameters constrained
*wR*(*F*^2^) = 0.076	*w* = 1/[σ^2^(*F*_o_^2^) + (0.0488*P*)^2^] where *P* = (*F*_o_^2^ + 2*F*_c_^2^)/3
*S* = 1.00	(Δ/σ)_max_ < 0.001
4775 reflections	Δρ_max_ = 0.23 e Å^−^^3^
343 parameters	Δρ_min_ = −0.30 e Å^−^^3^
1 restraint	Absolute structure: Flack (1983), 2006 Friedel pairs
Primary atom site location: structure-invariant direct methods	Flack parameter: 0.007 (7)

### Special details

**Table:**

Geometry. All e.s.d.'s (except the e.s.d. in the dihedral angle between two l.s. planes)are estimated using the full covariance matrix. The cell e.s.d.'s are takeninto account individually in the estimation of e.s.d.'s in distances, anglesand torsion angles; correlations between e.s.d.'s in cell parameters are onlyused when they are defined by crystal symmetry. An approximate (isotropic)treatment of cell e.s.d.'s is used for estimating e.s.d.'s involving l.s. planes.
Refinement. Refinement of *F*^2^ against ALL reflections. The weighted *R*-factor *wR* and goodness of fit *S* are based on *F*^2^, conventional *R*-factors *R* are based on *F*, with *F* set to zero for negative *F*^2^. The threshold expression of *F*^2^ > σ(*F*^2^) is used only for calculating *R*-factors(gt) *etc*. and is not relevant to the choice of reflections for refinement. *R*-factors based on *F*^2^ are statistically about twice as large as those based on *F*, and *R*- factors based on ALL data will be even larger.

### Fractional atomic coordinates and isotropic or equivalent isotropic displacement parameters (Å^2^)

**Table:**

	*x*	*y*	*z*	*U*_iso_*/*U*_eq_	
Br1	0.21282 (3)	0.79871 (5)	0.29748 (2)	0.05470 (12)	
Br2	−0.04494 (4)	0.42942 (6)	0.31148 (2)	0.06496 (14)	
C1	0.4329 (3)	0.2381 (4)	0.3577 (2)	0.0400 (8)	
C2	0.4454 (3)	0.1460 (5)	0.4312 (2)	0.0484 (9)	
H2	0.3732	0.1084	0.4434	0.058*	
C3	0.5632 (4)	0.1100 (5)	0.4859 (2)	0.0594 (11)	
H3	0.5697	0.0484	0.5346	0.071*	
C4	0.6707 (4)	0.1645 (6)	0.4690 (3)	0.0624 (11)	
H4	0.7498	0.1392	0.5060	0.075*	
C5	0.6613 (4)	0.2561 (5)	0.3976 (2)	0.0587 (11)	
H5	0.7342	0.2928	0.3860	0.070*	
C6	0.5430 (3)	0.2944 (6)	0.3424 (2)	0.0498 (8)	
H6	0.5375	0.3587	0.2947	0.060*	
C7	0.3069 (3)	0.2758 (5)	0.29839 (19)	0.0396 (7)	
C8	0.2064 (3)	0.3218 (5)	0.3282 (2)	0.0430 (8)	
H8	0.2173	0.3294	0.3852	0.052*	
C9	0.0902 (3)	0.3555 (4)	0.2707 (2)	0.0427 (9)	
C10	0.0688 (3)	0.3457 (4)	0.1861 (2)	0.0407 (8)	
H10	−0.0110	0.3693	0.1496	0.049*	
C11	0.1686 (3)	0.2998 (5)	0.15494 (18)	0.0350 (6)	
C12	0.2857 (3)	0.2664 (4)	0.21181 (19)	0.0387 (8)	
H12	0.3527	0.2366	0.1919	0.046*	
C13	0.1478 (3)	0.2884 (5)	0.06339 (18)	0.0354 (7)	
C14	0.2206 (3)	0.1799 (4)	0.0303 (2)	0.0389 (7)	
H14	0.2843	0.1142	0.0661	0.047*	
C15	0.1998 (3)	0.1679 (5)	−0.0555 (2)	0.0443 (8)	
H15	0.2501	0.0958	−0.0765	0.053*	
C16	0.1048 (3)	0.2626 (5)	−0.1094 (2)	0.0443 (9)	
H16	0.0896	0.2526	−0.1668	0.053*	
C17	0.0325 (3)	0.3720 (5)	−0.0781 (2)	0.0451 (9)	
H17	−0.0309	0.4375	−0.1143	0.054*	
C18	0.0537 (3)	0.3850 (4)	0.0076 (2)	0.0420 (8)	
H18	0.0041	0.4595	0.0281	0.050*	
C19	0.3980 (3)	0.7771 (4)	0.03861 (19)	0.0349 (7)	
C20	0.4802 (3)	0.8546 (4)	0.0009 (2)	0.0390 (8)	
H20	0.5522	0.9104	0.0338	0.047*	
C21	0.4564 (3)	0.8499 (4)	−0.0852 (2)	0.0442 (9)	
H21	0.5125	0.9019	−0.1094	0.053*	
C22	0.3502 (3)	0.7685 (5)	−0.1344 (2)	0.0452 (8)	
H22	0.3344	0.7654	−0.1920	0.054*	
C23	0.2669 (3)	0.6914 (4)	−0.0988 (2)	0.0431 (8)	
H23	0.1948	0.6369	−0.1324	0.052*	
C24	0.2907 (3)	0.6950 (4)	−0.0130 (2)	0.0399 (8)	
H24	0.2344	0.6420	0.0107	0.048*	
C25	0.4238 (3)	0.7824 (4)	0.13106 (18)	0.0364 (7)	
C26	0.3245 (3)	0.7809 (5)	0.16629 (19)	0.0390 (7)	
H26	0.2412	0.7743	0.1321	0.047*	
C27	0.3501 (3)	0.7894 (5)	0.25135 (19)	0.0408 (7)	
C28	0.4727 (3)	0.7964 (5)	0.30482 (18)	0.0427 (7)	
H28	0.4875	0.8016	0.3623	0.051*	
C29	0.5735 (3)	0.7955 (5)	0.27097 (18)	0.0383 (7)	
C30	0.5467 (3)	0.7882 (5)	0.18430 (18)	0.0383 (7)	
H30	0.6135	0.7871	0.1614	0.046*	
C31	0.7054 (3)	0.8081 (5)	0.32689 (19)	0.0407 (7)	
C32	0.7968 (3)	0.9055 (5)	0.3053 (2)	0.0488 (9)	
H32	0.7755	0.9609	0.2538	0.059*	
C33	0.9172 (3)	0.9211 (6)	0.3584 (3)	0.0634 (11)	
H33	0.9761	0.9888	0.3433	0.076*	
C34	0.9512 (4)	0.8367 (6)	0.4342 (3)	0.0693 (13)	
H34	1.0332	0.8467	0.4701	0.083*	
C35	0.8638 (4)	0.7381 (6)	0.4565 (3)	0.0625 (11)	
H35	0.8865	0.6809	0.5075	0.075*	
C36	0.7424 (4)	0.7236 (5)	0.4036 (2)	0.0515 (9)	
H36	0.6841	0.6561	0.4194	0.062*	

### Atomic displacement parameters (Å^2^)

**Table:**

	*U*^11^	*U*^22^	*U*^33^	*U*^12^	*U*^13^	*U*^23^
Br1	0.0503 (2)	0.0711 (3)	0.0487 (2)	−0.0041 (2)	0.02380 (16)	−0.0006 (2)
Br2	0.0576 (2)	0.0860 (3)	0.0622 (3)	0.0117 (2)	0.0346 (2)	−0.0023 (2)
C1	0.0496 (19)	0.0374 (18)	0.0336 (18)	0.0041 (14)	0.0131 (16)	−0.0058 (13)
C2	0.057 (2)	0.050 (2)	0.039 (2)	0.0019 (18)	0.0156 (18)	−0.0019 (16)
C3	0.076 (3)	0.056 (3)	0.039 (2)	0.011 (2)	0.007 (2)	−0.0004 (18)
C4	0.054 (2)	0.071 (3)	0.052 (2)	0.007 (2)	0.001 (2)	−0.010 (2)
C5	0.046 (2)	0.071 (3)	0.055 (2)	0.0002 (19)	0.0093 (19)	−0.008 (2)
C6	0.053 (2)	0.053 (2)	0.0430 (19)	0.001 (2)	0.0147 (16)	−0.003 (2)
C7	0.0467 (18)	0.035 (2)	0.0392 (17)	−0.0043 (16)	0.0157 (15)	−0.0042 (15)
C8	0.0509 (19)	0.044 (2)	0.0390 (17)	−0.0037 (17)	0.0204 (16)	−0.0053 (16)
C9	0.046 (2)	0.043 (2)	0.048 (2)	0.0015 (14)	0.0255 (17)	−0.0040 (15)
C10	0.0348 (16)	0.042 (2)	0.046 (2)	−0.0021 (13)	0.0127 (15)	−0.0006 (14)
C11	0.0390 (16)	0.0285 (15)	0.0383 (16)	0.0006 (16)	0.0130 (13)	−0.0014 (15)
C12	0.0390 (17)	0.040 (2)	0.0390 (18)	0.0007 (14)	0.0152 (15)	−0.0003 (15)
C13	0.0344 (15)	0.0317 (16)	0.0408 (17)	−0.0086 (15)	0.0120 (13)	−0.0023 (16)
C14	0.0376 (17)	0.0400 (19)	0.0378 (18)	0.0032 (14)	0.0093 (15)	−0.0008 (15)
C15	0.0437 (19)	0.047 (2)	0.045 (2)	−0.0006 (17)	0.0181 (17)	−0.0066 (17)
C16	0.0475 (19)	0.051 (2)	0.0343 (18)	−0.0076 (16)	0.0120 (15)	0.0008 (16)
C17	0.0415 (19)	0.050 (2)	0.039 (2)	−0.0005 (15)	0.0043 (16)	0.0077 (15)
C18	0.0348 (17)	0.046 (2)	0.044 (2)	0.0011 (15)	0.0105 (16)	−0.0012 (16)
C19	0.0345 (15)	0.0327 (18)	0.0365 (16)	0.0045 (14)	0.0090 (13)	−0.0003 (15)
C20	0.0361 (17)	0.0402 (19)	0.0397 (19)	−0.0039 (13)	0.0101 (15)	−0.0033 (14)
C21	0.049 (2)	0.045 (2)	0.042 (2)	0.0008 (15)	0.0178 (17)	−0.0013 (15)
C22	0.0492 (19)	0.051 (2)	0.0331 (17)	0.0039 (17)	0.0098 (15)	−0.0032 (16)
C23	0.0400 (18)	0.047 (2)	0.0370 (19)	−0.0033 (16)	0.0034 (15)	−0.0100 (16)
C24	0.0369 (17)	0.041 (2)	0.0422 (19)	−0.0048 (14)	0.0129 (15)	−0.0010 (15)
C25	0.0424 (16)	0.0324 (18)	0.0341 (16)	0.0006 (15)	0.0108 (13)	−0.0039 (15)
C26	0.0380 (16)	0.0408 (19)	0.0377 (17)	−0.0023 (15)	0.0109 (13)	−0.0021 (16)
C27	0.0452 (18)	0.0381 (18)	0.0427 (18)	−0.0019 (17)	0.0185 (15)	0.0014 (17)
C28	0.0517 (19)	0.0433 (18)	0.0331 (17)	0.0034 (19)	0.0129 (15)	0.0026 (18)
C29	0.0411 (16)	0.0335 (16)	0.0376 (16)	0.0008 (16)	0.0079 (13)	−0.0056 (16)
C30	0.0373 (17)	0.0397 (18)	0.0393 (17)	0.0037 (16)	0.0138 (14)	0.0002 (18)
C31	0.0447 (18)	0.0406 (19)	0.0341 (17)	0.0114 (19)	0.0078 (14)	−0.0039 (18)
C32	0.0439 (19)	0.051 (2)	0.048 (2)	0.0030 (18)	0.0086 (17)	0.0031 (18)
C33	0.044 (2)	0.066 (3)	0.074 (3)	−0.002 (2)	0.008 (2)	−0.012 (3)
C34	0.049 (2)	0.075 (3)	0.065 (3)	0.016 (2)	−0.012 (2)	−0.022 (2)
C35	0.068 (3)	0.067 (3)	0.044 (2)	0.017 (2)	0.003 (2)	−0.0024 (19)
C36	0.055 (2)	0.054 (2)	0.039 (2)	0.0107 (18)	0.0048 (18)	−0.0012 (17)

### Geometric parameters (Å, °)

**Table:**

Br1—C27	1.903 (3)	C18—H18	0.9300
Br2—C9	1.911 (3)	C19—C20	1.391 (4)
C1—C6	1.389 (5)	C19—C24	1.397 (5)
C1—C2	1.395 (5)	C19—C25	1.491 (4)
C1—C7	1.480 (5)	C20—C21	1.388 (5)
C2—C3	1.381 (5)	C20—H20	0.9300
C2—H2	0.9300	C21—C22	1.372 (5)
C3—C4	1.371 (6)	C21—H21	0.9300
C3—H3	0.9300	C22—C23	1.377 (5)
C4—C5	1.370 (6)	C22—H22	0.9300
C4—H4	0.9300	C23—C24	1.385 (5)
C5—C6	1.391 (5)	C23—H23	0.9300
C5—H5	0.9300	C24—H24	0.9300
C6—H6	0.9300	C25—C30	1.387 (4)
C7—C12	1.402 (4)	C25—C26	1.396 (4)
C7—C8	1.396 (4)	C26—C27	1.371 (4)
C8—C9	1.382 (5)	C26—H26	0.9300
C8—H8	0.9300	C27—C28	1.386 (4)
C9—C10	1.370 (5)	C28—C29	1.397 (4)
C10—C11	1.403 (4)	C28—H28	0.9300
C10—H10	0.9300	C29—C30	1.395 (4)
C11—C12	1.385 (4)	C29—C31	1.484 (4)
C11—C13	1.486 (4)	C30—H30	0.9300
C12—H12	0.9300	C31—C32	1.394 (5)
C13—C14	1.391 (4)	C31—C36	1.392 (5)
C13—C18	1.392 (5)	C32—C33	1.370 (5)
C14—C15	1.391 (5)	C32—H32	0.9300
C14—H14	0.9300	C33—C34	1.378 (6)
C15—C16	1.376 (5)	C33—H33	0.9300
C15—H15	0.9300	C34—C35	1.371 (6)
C16—C17	1.375 (5)	C34—H34	0.9300
C16—H16	0.9300	C35—C36	1.375 (5)
C17—C18	1.389 (5)	C35—H35	0.9300
C17—H17	0.9300	C36—H36	0.9300
			
C6—C1—C2	117.6 (3)	C20—C19—C24	117.8 (3)
C6—C1—C7	121.2 (3)	C20—C19—C25	120.9 (3)
C2—C1—C7	121.2 (3)	C24—C19—C25	121.3 (3)
C3—C2—C1	121.0 (4)	C21—C20—C19	121.1 (3)
C3—C2—H2	119.5	C21—C20—H20	119.5
C1—C2—H2	119.5	C19—C20—H20	119.5
C4—C3—C2	120.4 (4)	C22—C21—C20	119.9 (3)
C4—C3—H3	119.8	C22—C21—H21	120.0
C2—C3—H3	119.8	C20—C21—H21	120.0
C5—C4—C3	119.8 (4)	C21—C22—C23	120.3 (3)
C5—C4—H4	120.1	C21—C22—H22	119.9
C3—C4—H4	120.1	C23—C22—H22	119.9
C4—C5—C6	120.1 (4)	C22—C23—C24	119.9 (3)
C4—C5—H5	119.9	C22—C23—H23	120.0
C6—C5—H5	119.9	C24—C23—H23	120.0
C1—C6—C5	121.0 (4)	C23—C24—C19	121.0 (3)
C1—C6—H6	119.5	C23—C24—H24	119.5
C5—C6—H6	119.5	C19—C24—H24	119.5
C12—C7—C8	118.7 (3)	C30—C25—C26	118.3 (3)
C12—C7—C1	121.1 (3)	C30—C25—C19	121.0 (3)
C8—C7—C1	120.2 (3)	C26—C25—C19	120.7 (3)
C9—C8—C7	118.3 (3)	C27—C26—C25	119.8 (3)
C9—C8—H8	120.8	C27—C26—H26	120.1
C7—C8—H8	120.8	C25—C26—H26	120.1
C10—C9—C8	123.2 (3)	C26—C27—C28	122.2 (3)
C10—C9—Br2	118.5 (3)	C26—C27—Br1	118.9 (2)
C8—C9—Br2	118.2 (2)	C28—C27—Br1	118.8 (2)
C9—C10—C11	119.3 (3)	C27—C28—C29	119.0 (3)
C9—C10—H10	120.3	C27—C28—H28	120.5
C11—C10—H10	120.3	C29—C28—H28	120.5
C12—C11—C10	118.1 (3)	C30—C29—C28	118.5 (3)
C12—C11—C13	121.6 (3)	C30—C29—C31	121.5 (3)
C10—C11—C13	120.3 (3)	C28—C29—C31	119.9 (3)
C11—C12—C7	122.4 (3)	C25—C30—C29	122.2 (3)
C11—C12—H12	118.8	C25—C30—H30	118.9
C7—C12—H12	118.8	C29—C30—H30	118.9
C14—C13—C18	117.6 (3)	C32—C31—C36	117.3 (3)
C14—C13—C11	121.3 (3)	C32—C31—C29	121.7 (3)
C18—C13—C11	121.1 (3)	C36—C31—C29	121.0 (3)
C13—C14—C15	121.1 (3)	C33—C32—C31	121.4 (4)
C13—C14—H14	119.4	C33—C32—H32	119.3
C15—C14—H14	119.4	C31—C32—H32	119.3
C16—C15—C14	120.1 (3)	C32—C33—C34	120.1 (4)
C16—C15—H15	119.9	C32—C33—H33	119.9
C14—C15—H15	119.9	C34—C33—H33	119.9
C17—C16—C15	119.7 (3)	C35—C34—C33	119.7 (4)
C17—C16—H16	120.1	C35—C34—H34	120.2
C15—C16—H16	120.1	C33—C34—H34	120.2
C16—C17—C18	120.2 (3)	C34—C35—C36	120.3 (4)
C16—C17—H17	119.9	C34—C35—H35	119.9
C18—C17—H17	119.9	C36—C35—H35	119.9
C17—C18—C13	121.1 (3)	C35—C36—C31	121.2 (4)
C17—C18—H18	119.4	C35—C36—H36	119.4
C13—C18—H18	119.4	C31—C36—H36	119.4
			
C6—C1—C2—C3	−1.1 (5)	C24—C19—C20—C21	−0.2 (5)
C7—C1—C2—C3	179.5 (3)	C25—C19—C20—C21	180.0 (3)
C1—C2—C3—C4	0.0 (6)	C19—C20—C21—C22	0.3 (5)
C2—C3—C4—C5	0.4 (6)	C20—C21—C22—C23	0.0 (5)
C3—C4—C5—C6	0.2 (6)	C21—C22—C23—C24	−0.4 (5)
C2—C1—C6—C5	1.7 (5)	C22—C23—C24—C19	0.4 (5)
C7—C1—C6—C5	−178.9 (4)	C20—C19—C24—C23	−0.2 (5)
C4—C5—C6—C1	−1.3 (6)	C25—C19—C24—C23	179.6 (3)
C6—C1—C7—C12	40.4 (5)	C20—C19—C25—C30	−30.1 (5)
C2—C1—C7—C12	−140.2 (3)	C24—C19—C25—C30	150.1 (3)
C6—C1—C7—C8	−139.7 (4)	C20—C19—C25—C26	150.2 (3)
C2—C1—C7—C8	39.8 (5)	C24—C19—C25—C26	−29.6 (5)
C12—C7—C8—C9	0.1 (5)	C30—C25—C26—C27	1.6 (5)
C1—C7—C8—C9	−179.8 (3)	C19—C25—C26—C27	−178.7 (3)
C7—C8—C9—C10	0.2 (5)	C25—C26—C27—C28	−1.2 (6)
C7—C8—C9—Br2	−176.9 (3)	C25—C26—C27—Br1	176.9 (3)
C8—C9—C10—C11	−0.2 (5)	C26—C27—C28—C29	0.3 (6)
Br2—C9—C10—C11	176.9 (2)	Br1—C27—C28—C29	−177.8 (3)
C9—C10—C11—C12	−0.1 (5)	C27—C28—C29—C30	0.2 (5)
C9—C10—C11—C13	−179.9 (3)	C27—C28—C29—C31	178.2 (4)
C10—C11—C12—C7	0.5 (5)	C26—C25—C30—C29	−1.1 (5)
C13—C11—C12—C7	−179.7 (3)	C19—C25—C30—C29	179.1 (3)
C8—C7—C12—C11	−0.4 (5)	C28—C29—C30—C25	0.3 (6)
C1—C7—C12—C11	179.5 (3)	C31—C29—C30—C25	−177.7 (3)
C12—C11—C13—C14	27.4 (5)	C30—C29—C31—C32	37.1 (5)
C10—C11—C13—C14	−152.8 (3)	C28—C29—C31—C32	−140.9 (4)
C12—C11—C13—C18	−153.2 (3)	C30—C29—C31—C36	−143.4 (4)
C10—C11—C13—C18	26.6 (5)	C28—C29—C31—C36	38.6 (5)
C18—C13—C14—C15	−0.2 (5)	C36—C31—C32—C33	−1.7 (6)
C11—C13—C14—C15	179.2 (3)	C29—C31—C32—C33	177.8 (4)
C13—C14—C15—C16	−0.8 (5)	C31—C32—C33—C34	1.4 (6)
C14—C15—C16—C17	1.5 (5)	C32—C33—C34—C35	−0.5 (6)
C15—C16—C17—C18	−1.0 (5)	C33—C34—C35—C36	−0.2 (6)
C16—C17—C18—C13	0.0 (5)	C34—C35—C36—C31	−0.2 (6)
C14—C13—C18—C17	0.6 (5)	C32—C31—C36—C35	1.1 (5)
C11—C13—C18—C17	−178.7 (3)	C29—C31—C36—C35	−178.5 (3)

### Hydrogen-bond geometry (Å, °)

**Table:**

*D*—H···*A*	*D*—H	H···*A*	*D*···*A*	*D*—H···*A*
C15—H15···Cg1^i^	0.93	2.82	3.601 (4)	142
C18—H18···Cg6^ii^	0.93	2.84	3.682 (4)	152
C20—H20···Cg1^iii^	0.93	2.92	3.603 (4)	132

Symmetry codes: (i) *x*, *y*−1, *z*; (ii) −*x*, *y*+1/2, −*z*; (iii) −*x*+1, *y*+1/2, −*z*.
